# Hormonal Dynamics in Response to Short-Term Hypoxia Exposure: Cortisol and Prolactin Regulation as Adaptive Mechanisms

**DOI:** 10.7759/cureus.72703

**Published:** 2024-10-30

**Authors:** Lana Ruzic, Girgis Kalim Assaf, Vojko Vuckovic, Maja Cigrovski Berkovic

**Affiliations:** 1 Department of Sport and Exercise Medicine, Faculty of Kinesiology, University of Zagreb, Zagreb, HRV; 2 Department of Sport, Antonine University, Baabda, LBN; 3 Department of Sport, Faculty of Sport, University of Ljubljana, Ljubljana, SVN

**Keywords:** altitude simulation, hormone response, hypoxia rooms, managers, stress

## Abstract

Background: The better availability of altitude rooms in hotels and sports centers led to increased people speculating that “over-the-weekend” hypoxia might contribute to overall psychological and physical well-being.

Purpose: This study investigates the acute stress hormone response in management professionals following short-term exposure to normobaric hypoxia, amidst the increasing use of hypoxic conditions in stress-relief programs, which lack scientific validation.

Methods: Twelve healthy male subjects, employees in management positions (mean age 34.72±7.43y), voluntarily agreed to participate in the within-subjects design study and were subjected to sleep and stay in normobaric hypoxia (simulating oxygen pressure at 2800m) twice, for at least 12h per exposure, over a total period of 48 hours to simulate popular “manager weekend hypoxia stays”. The protocol included blood sampling 48 hours before intervention, on the morning of intervention, and immediately after finishing the hypoxia stay. We determined the concentrations of total testosterone, dehydroepiandrosterone-sulfate (DHEA-S), prolactin, and cortisol.

Results: A significant increase in prolactin (p=0.021) and cortisol (p=0.039) concentrations and a decrease in DHEA-S to cortisol ratio (p=0.034) were observed.

Discussion and conclusion: Short exposure to normobaric hypoxia induces alterations in the hypothalamic-pituitary-adrenal and pituitary-gonadal axis showing significant stress response even though otherwise was expected due to participants’ stressful jobs.

## Introduction

Mild hypoxia can influence decision-making processes and increase a person’s ability to participate in riskier actions. Hypoxic conditioning by recurrent exposure to hypoxia has been suggested to have non-pharmacological therapeutic benefits in terms of enhancing the physiological functions of individuals in whom acute or chronic pathological events are either anticipated or already existing. Several animal experimental studies suggested repetitive exposure to hypoxia might induce even more prolonged and sustained protection. It seems that mild hypoxia can induce more risky behavior, either under known or ambiguous risk [1,2]. The human body adapts to hypoxia, depending on the exposure time and degree by different mechanisms in the homeostatic state of metabolic functions but also in endocrine functions [3,4].

Activation of the hypothalamic-pituitary-adrenocortical (HPA) axis represents a primary endocrine response to stress [5]. The HPA axis, through the release of cortisol, initiates a catabolic state and mobilizes energy reserve for the immediate danger, but also participates in the preparation for predicted insult, a so-called anticipatory response [6]. Elevated blood cortisol levels have been observed in critically ill medical and surgical patients and are correlated with worse outcomes. Increased cortisol levels may cause the reduction of free testosterone (fT), and some types of physical preparation causing cortisol elevation have been negatively associated with total testosterone or dehydroepiandrosterone-sulfate (DHEA-S). In persons in managing positions, the interplay between cortisol and testosterone might be the key predictor of social status and success [7,8]. On the other hand, DHEA-S, another steroid hormone from the adrenal cortex, might counterbalance the negative effects of cortisol and lead to anabolic and anti-inflammatory effects. The DHEA-S and cortisol ratio appears to have an important health role. An altered ratio (favoring cortisol increase and DHEA-S decrease) is characteristic of the age-related loss of immunity, higher anxiety, mood disturbance, confusion, and poorer cognitive performance [9].

The better availability of altitude rooms in many hotels and sports centers led to an increase in the number of people speculating that “over-the-weekend” altitude hypoxia simulation might contribute to overall psychological and physical well-being. Still, this normobaric hypoxia might influence the concentration of stress hormones like the altitude-induced stress response. Acute stress has some benefits as it may trigger adaptation processes and may lead to better future stress resilience. Additionally, it might also have some negative effects (like in immunity while suppressing macrophage mobility and IL-6 concentrations) especially if there is no recovery period and, in the case, when usual work-related stress continues immediately after [10]. 

Reports on the effects of hypoxic exposure on hormones are limited, often contradictory, and rarely involve non-athletes. With overnight stays in hypoxic conditions becoming more financially feasible and accessible, an increasing number of non-athletes are choosing to participate, as many facilities include hypoxic exposure in their stress-relief programs. However, the scientific validity of these programs in providing stress relief has not been confirmed. Therefore, this study investigates the acute stress hormone response following short-term exposure to normobaric hypoxia in individuals in management positions.

## Materials and methods

Study design

The experiment used a within-subject design to determine the effects of short-term normobaric hypoxia on hormonal responses. The effects were controlled by comparing the pre/post scores with no intervention to the pre/post scores of the same subject in intervention conditions. Each subject served as his control in no intervention/intervention conditions. We hypothesized that relaxation and spa-hypoxia rooms might contribute to the stress lowering and decrease in stress hormones after a week of work. That is why the first condition was during the week, in the usual work environment, and the second condition i.e. hypoxia was during the weekend. All other factors like meals and levels of physical activity (physical exercise) were allowed up to three days before the beginning of the study while during the study the subjects were instructed not to perform any physical exertion above anaerobic threshold like training and similar. In addition, confrontations with friends, family, and coworkers were monitored to keep those as alike as possible during the two conditions. All those factors were discussed in an interview between the main investigator and the subjects before each measurement. Moreover, we monitored participants’ behavior and basic motor abilities one hour apart from taking blood samples by simple motor tests lasting less than one minute, performed on three occasions [11].

All subjects signed an informed consent form after they were introduced in an oral and written manner to the study plan, potential risks, and benefits. The study was approved by the Ethics Committee of the Faculty of Kinesiology, University of Zagreb, Croatia (Approval No 27/2021 on 27/01/2021).

Subjects and methods

Twelve male subjects (mean age 34.72±7.43 years) voluntarily agreed to participate in the study. They were all employees in management positions with demanding jobs requiring daily customer or coworker contacts and conversations. All were healthy, with no known chronic condition that would require the use of medications.

During the first part of the study (between the first and second measurement), and then between the second and the third measurement participants performed activities of their choice, at low intensity. During the two nights of intervention (simulation of “manager weekend hypoxia stay”), they stayed (and slept) in normobaric hypoxia twice (due to the study goal set oxygen was as at 2800m, i.e. about 14.5% of the so-called effective oxygen), for at least 12 hours per session (8 pm to 8 am), over a total period of 48 hours. The intervention was simulated in Planica Nordic Centre, Slovenia. Peripheral blood oxygen saturation (SpO2) was monitored at least three times during the stay in the hypoxia room with Beurer Po45 oximeters (Beurer GmbH, Ulm, Germany). Values collected one hour after entering the hypoxia rooms, before going to sleep, and after waking up were transcribed and further analyzed.

**Figure 1:**
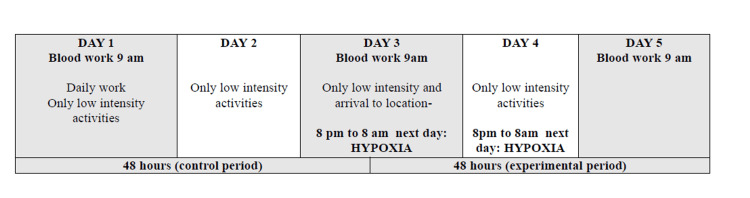
Study protocol for subjects utilizing cross-over design

The protocol presented in Figure 1 involved blood sampling on three occasions: 48 hours before the day of intervention (Wednesday), at the beginning of intervention (Friday), and immediately after finishing the hypoxia stay (Sunday at the location of the intervention). Blood samples were taken in the morning (every sampling at 9 am) after a night's sleep and 30 minutes rest in a dark room, on an empty stomach. Concentrations of total testosterone, DHEA-S, prolactin, and cortisol were measured by a licensed laboratory, BIOLAB, Ljubljana, Slovenia.

## Results

Participants' characteristics are presented in Table 1. Mean values of peripheral oxygen saturation were 92.8±1.68% one hour after entering the hypoxia room, then recovered to 94±1.63% probably due to ventilatory adaptations but dropped again to 92.7±1.88% the following morning.

**Table 1:** Participants' characteristics BMI: body mass index

	Mean	Min	Max	SD
Height (cm)	182.09	173.00	198.00	7.905
Weight (kg)	83.55	72.00	96.00	7.621
BMI (kg/m^2^)	25.27	20.92	28.73	2.607

Significant post-intervention changes were determined in two of the measured hormones; results are presented in Table 2.

**Table 2:** Changes in the hormone levels DHEA-S: dehydroepiandrosterone-sulfate

	Before 1	Before 2	After
Prolactin (mIU/L)	225.23±118.79	220.30±61.65	377.32±113.10
Testosterone total (mmol/L)	22.57±6.51	22.65±5.90	24.68±5.60
Cortisol (mmol/L)	369.47±113.10	379.43±105.39	451.88±69.76
DHEA-S (μmol/L)	8.69±4.14	8.73±3.68	8.94±3.89
DHEA-S/Cortisol ratio	0.023±0.01	0.023±0.010	0.018±0.007

**Figure 2:**
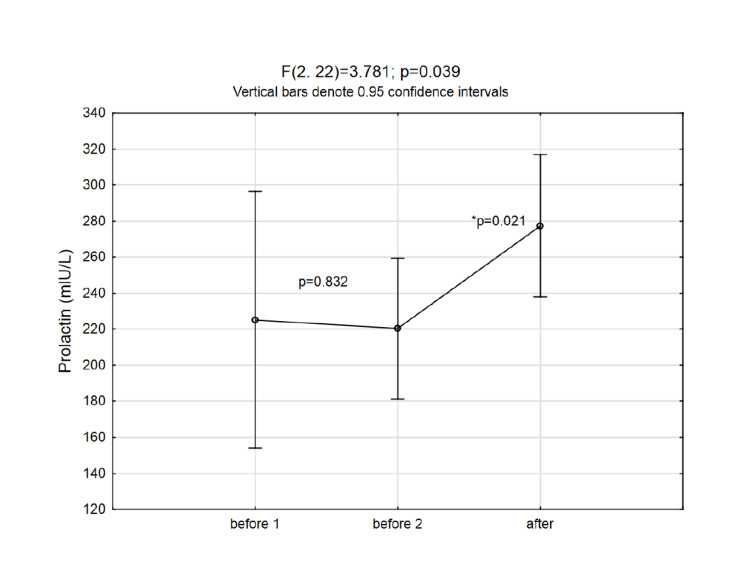
Changes in prolactin levels at three time points (p-values in the graph area are the post hoc Fisher LSD test results) LSD: least significant difference

**Figure 3:**
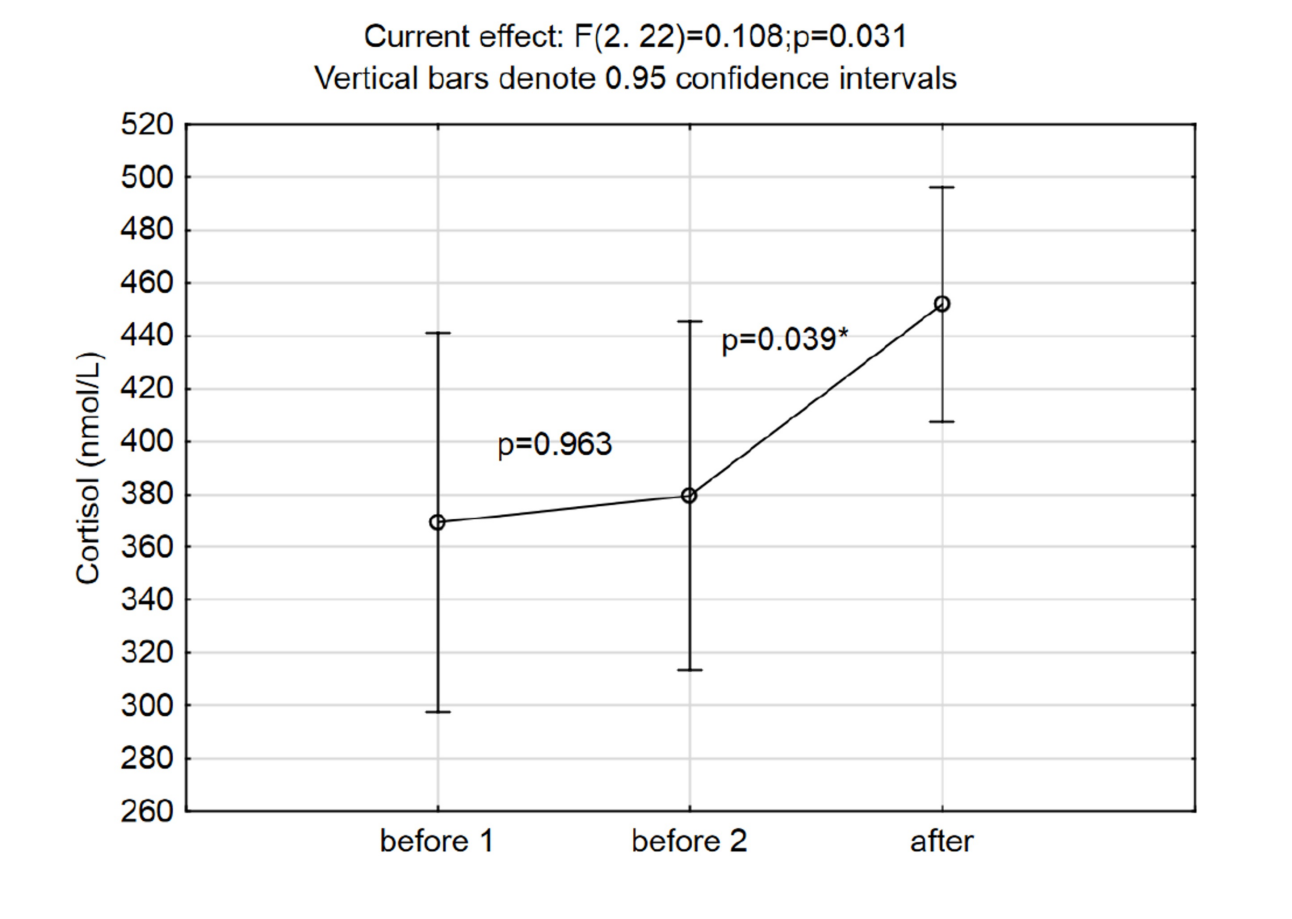
Changes in cortisol levels at three time points (p-values in the graph area are the post hoc Fisher LSD test results) LSD: least significant difference

As the subjects served as their controls, the time points are defined as BEFORE (time points before 1 and before 2) and AFTER (meaning after protocol) time points. The cortisol and prolactin concentrations were significantly higher immediately after intervention (ANOVA for repeated measures and post-hoc Fisher test results are presented in Figures 2, 3).

Due to the small sample size (which was to an extent overcome by the within design as subjects served as their controls), the post-hoc power analysis was performed in free G*Power software. For the significant change in prolactin levels after the intervention, the calculated Cohens d effect size was 0.920, which at alpha 0.05 and beta 0.20, gave a satisfactory obtained power of 0.909.

The Cohen d effect size for the change in cortisol levels after the hypoxia was 0.72 and obtained a power of 0.82.

**Figure 4:**
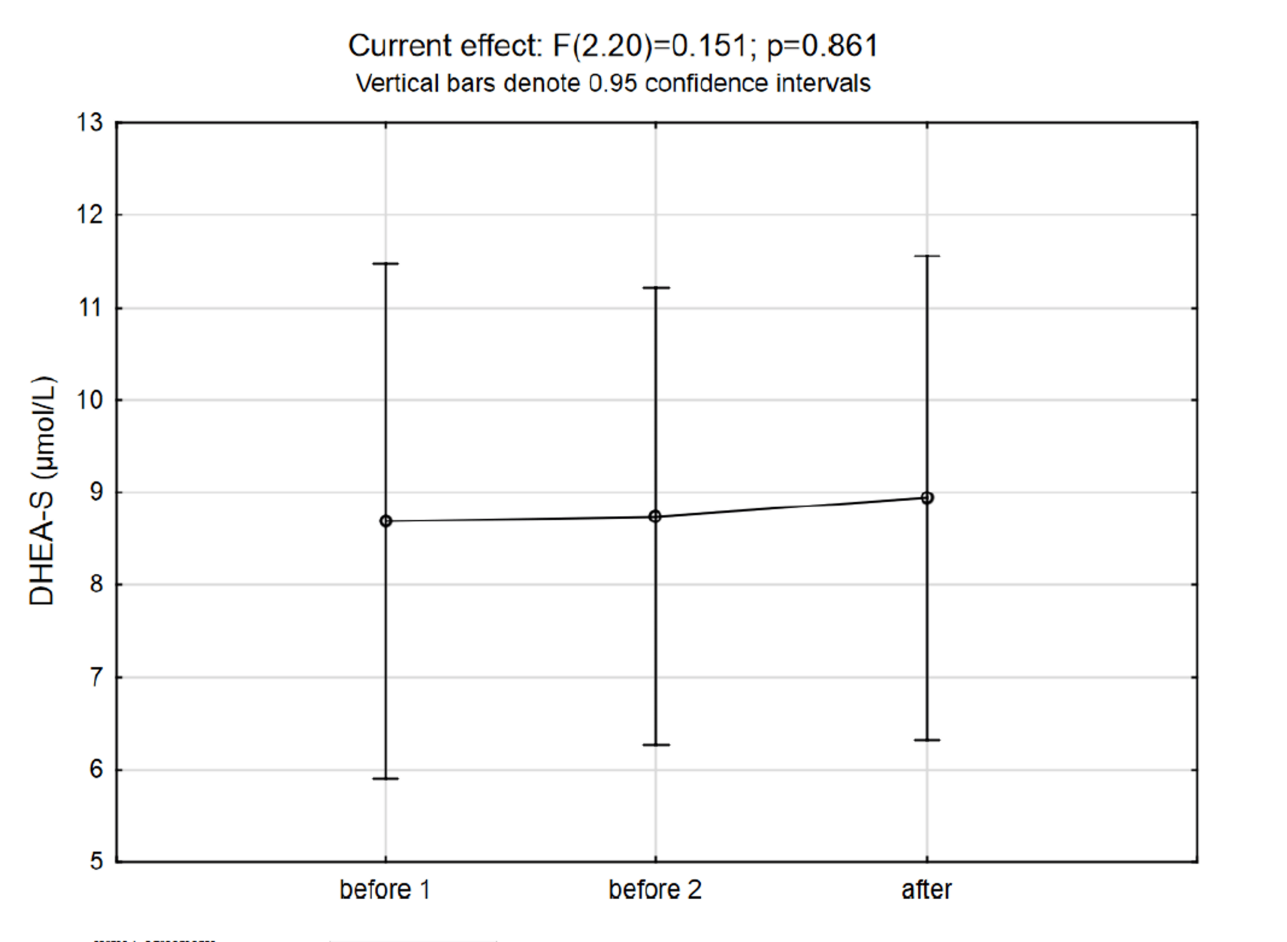
Changes in DHEA-S levels were not significant DHEA-S: dehydroepiandrosterone-sulfate

**Figure 5:**
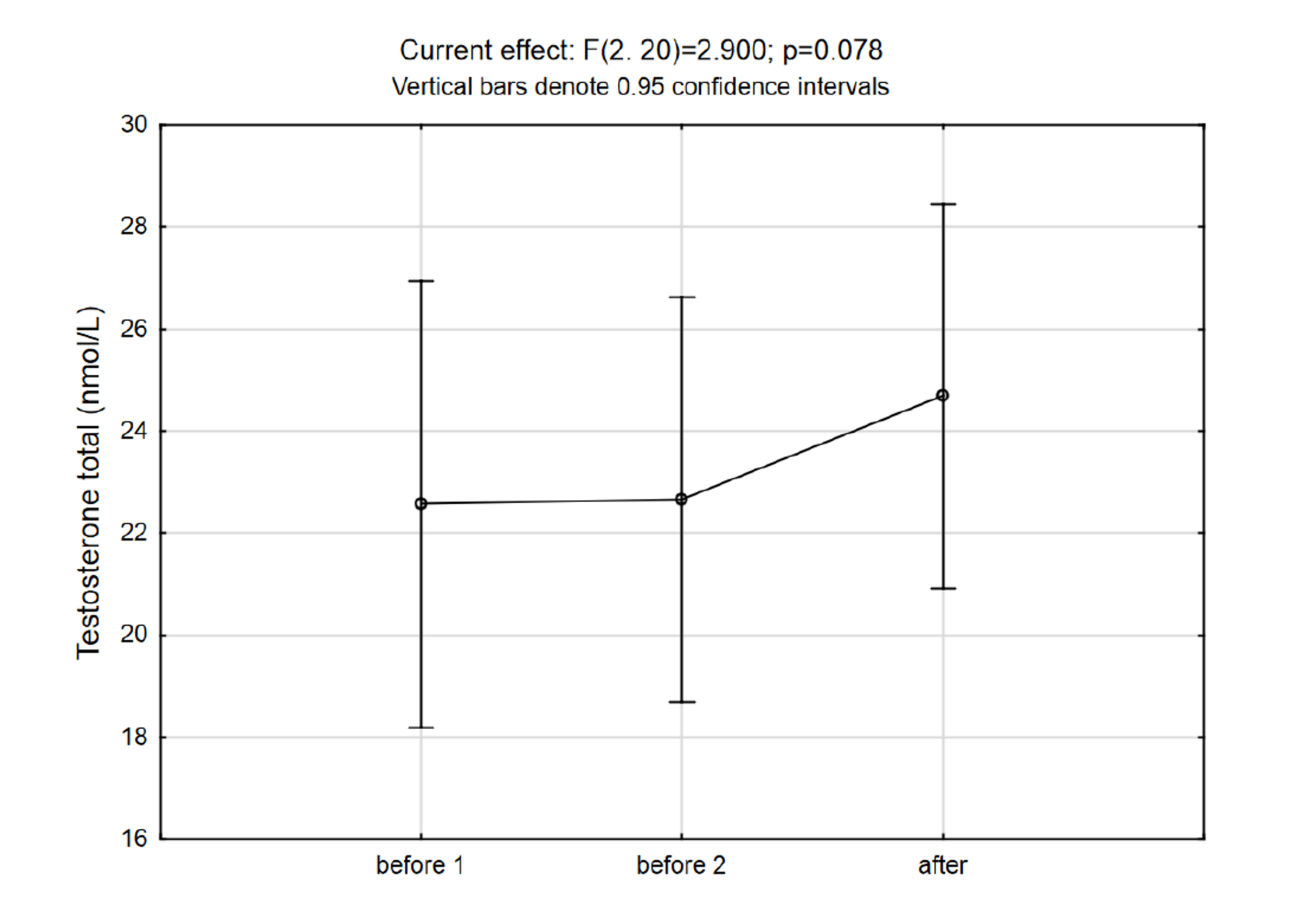
Testosterone levels show no significant change despite the increase after the intervention

**Figure 6:**
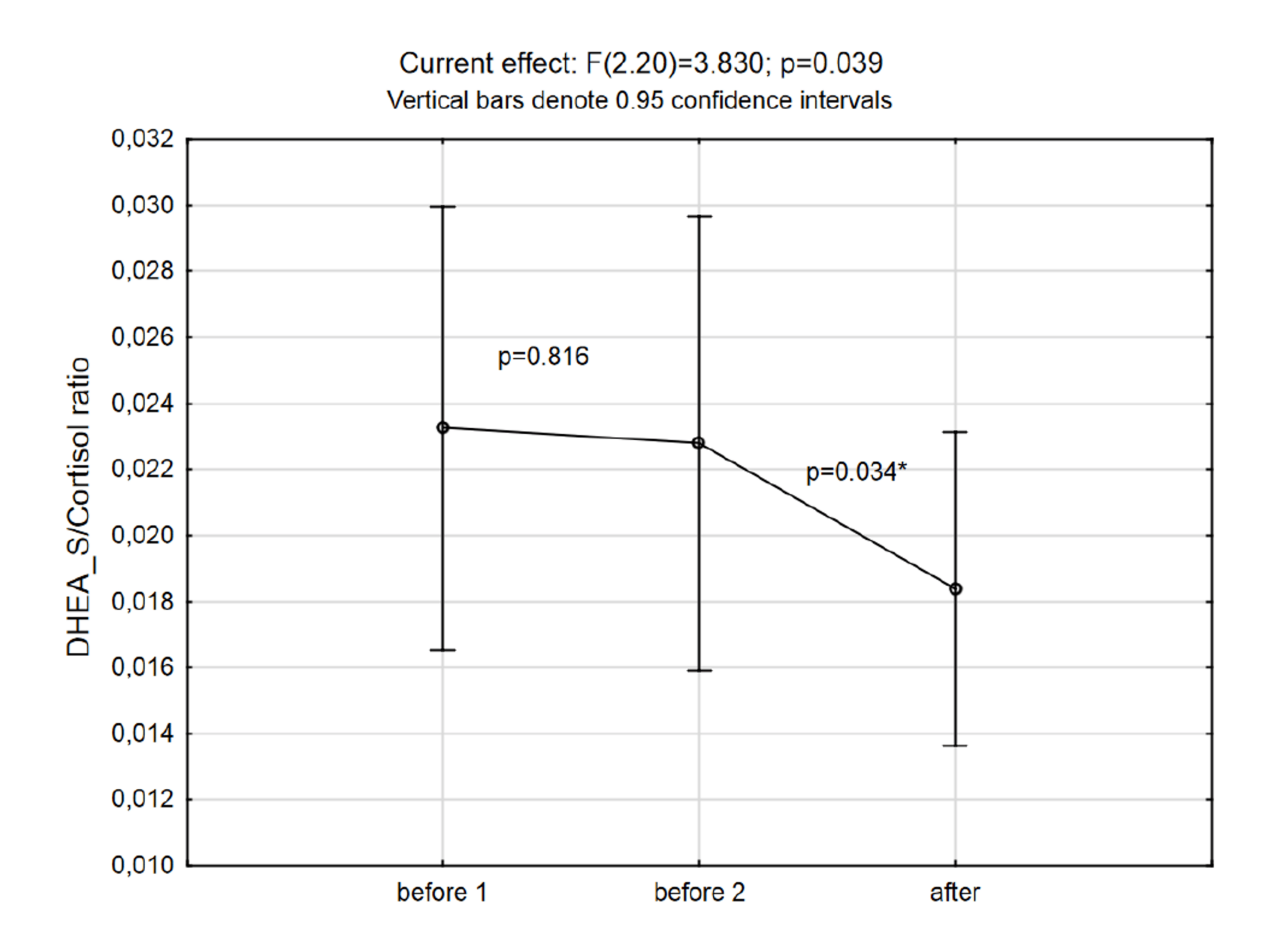
Changes in DHEA-S to cortisol ratio at three time points (p-values in the graph area are the post-hoc Fisher LSD test results) DHEA-S: dehydroepiandrosterone-sulfate; LSD: least significant difference

No significant changes after the intervention were observed in DHEA-S concentrations, while even though the total testosterone was elevated to some extent, the increase was not statistically significant (Figure 4 and Figure 5). The changes in DHEA-S to cortisol ratio are presented in Figure 6.

It is assumed that a person with a higher ratio (higher DHEA-S less cortisol) might suffer fewer negative effects from different stressors compared to a person with a lower ratio (less DHEA-S higher cortisol). Still, in this case, the obtained power was not satisfactory; therefore, conclusions should be drawn with consideration. The mean peripheral blood oxygen saturations during the stay in altitude rooms varied to a small extent among the subjects, ranging from 89 to 93% (data not shown).

## Discussion

Psychosocial stress is an important contributor to disease, and both repeated acute and chronic mental stress, as seen in persons in management positions, may affect health, quality of life, decision-making, and work productivity [12]. Therefore, finding different means of stress relief seems important from the preventive medicine aspect. Currently, exposure to hypoxia through a stay in hypoxic chambers which are nowadays readily available and marketed, is receiving popularity among managers as an “over the weekend hypoxia” wellness. Although the foundation for hypoxic (pre)conditioning is strong, the translation of different strategies into effective therapies and clinical practice is mainly missing.

Changes in the secretion of hormones while under the stress of normobaric hypoxic conditions are not univocal and have the potential for both beneficial and detrimental effects. The physiological stress response is mediated via the HPA axis [13]. A significant increase in cortisol and prolactin concentrations occurred, while the detected trend in testosterone rise did not reach statistical significance. The increase in the cortisol level after the hypoxia stay reached 451.88±69.76 nmol/L, comparable to that reported by Phillips and coworkers in a study including Vietnam veterans [14]. The observed decrease in SpO2, with the expected saturation at an altitude of 2800 m above sea level, was substantial enough to evoke a stress-hormonal response. It was shown previously that in patients with sleep apnea, the apnea index correlated with released cortisol [15].

DHEA, as opposed to cortisol, is a sex hormone precursor, and an anabolic steroid with a regenerative and immunoprotective role [16]. Therefore, DHEA secretion following acute stress was postulated to play a protective role as an antagonist of other stress hormones [17], and some studies reported a link between DHEA levels and stress intensity [18]. Moreover, DHEA is also a marker of mental stress, and it increases 1h following stress and progressively decreases after the stress ends [19]. In our study, DHEA-S levels remained unchanged when compared to pre-hypoxic levels. On the other hand, the DHEA-S to cortisol ratio seemed to decrease after the exposure to hypoxia suggesting that over the weekend wellness negatively affected stress rather than leading to its relief.

In addition, physiological and psychological stress affect prolactin release [20,21]. In our study, prolactin concentration increased. A similar adaptive response in prolactin rise following high altitude exposure was reported by Benso et al., who explained the mentioned finding by the neuroendocrine stimulation induced by stress [22]. 

Chronically stressed individuals, such as those with post-traumatic stress disorders, mainly showed an increase in prolactin and cortisol levels [23-25]. Marathon runners who are considered to be under significant level of stress also showed 40-50% higher prolactin levels than the average population [26]. 

Unlike other authors, we have found a trending rise in testosterone levels after short-term exposure to normobaric hypoxia, although it did not reach statistical significance. This would suggest the benefit of normobaric hypoxic chambers and the potential to induce anabolic effects and improvement of performance. Moreover, a rise in testosterone levels might also improve decision-making in management positions [7].

Underlying physiological mechanisms are complex. The HPA axis contributes to cortisol release as a response to mobilize energy sources in times of limited oxygen delivery to the brain and other organs. Prolactin is a stress hormone, but its response might be additionally stimulated in hypoxic conditions due to its involvement in angiogenesis [27]. Moreover, the release of hypoxia-inducible factor (HIF), responsible for cellular changes due to low SpO2, may also stimulate cortisol and prolactin release [28]. HIF-1α (as a transcription factor involved in signaling pathway playing a crucial role in cellular responses to low oxygen levels) is elevated whenever lungs are involved, such as with pleural effusion, so it might be expected that its involvement is a link between the short-term hypoxia, such as in our study, and stress response [29]. Still, the direct relation between HIF-1 α and cortisol and prolactin levels has not been sufficiently studied in humans.

The practical importance of the obtained results might be applied in a few areas. In leisure and tourism, the users might need to be warned about the stressful effects of hypoxia rooms in hotels, and in occupational medicine as the workers who are exposed to hypoxic environments may require stress hormone monitoring and might benefit from stress relieving methods. It is not clear at this point whether the decrease in strength, as measured also in the same set of patients [11] could be related to stress hormone levels but there might be some causal relations. It seems that longer exposure to stress hormones may provoke fatigue and decrease strength (even though it is very well known that acute response may increase strength in emergencies by recruiting more muscle fibers). 

We would like to emphasize that altitude rooms play a very significant role in athletes' training and preparation. Even in managers, like in this study, their value may be very well used if the goals are set. Sometimes it is important to provoke the stress to increase adaptability to future similar situations. Thus, these results should not at all be considered as "against“ altitude rooms; on the contrary, these results emphasize other possible applications, not only improvement of the oxygen transport system. The potential use of hypoxia rooms in occupational health may benefit workers preparing for high-altitude projects. While these rooms may induce some level of stress, they could aid in future adaptation to real conditions, especially for mining and construction workers who have not previously experienced high-altitude stress.

However, current knowledge is insufficient to provide strong recommendations for managers utilizing altitude rooms, and future research with larger sample sizes is necessary to mitigate potential negative effects. For now, we suggest using lower altitudes (such as 2000 m) and staying for longer than one weekend, as one factor contributing to the acute hormonal stress response may be the insufficient duration to elicit potential stress relief benefits.

At the same time, study limitations must be acknowledged. First of all, the sample size is small, and therefore, results cannot be generalized. In addition, stress self-perception was not objectively assessed, therefore, disabling the correlation of laboratory results with perceived stress in different study settings. 

## Conclusions

Our study demonstrates that exposure to hypoxia rooms over a weekend induces significant changes in cortisol and prolactin levels, suggesting a strong interaction of physiological mechanisms in response to reduced oxygen availability. Further studies are required to address the optimal hypoxic stimulus and the most favorable setting in which it can be applied to down-regulate psychological stress. This study underscores the intricate hormonal adaptations to hypoxia and highlights the practical implications for various fields, ranging from tourism, and high-altitude medicine to occupational health.
